# Supplementary material for: The major surface protein of malaria sporozoites is GPI-anchored to the plasma membrane
Source: J Biol Chem. 2024 Jul 11;300(8):107557. doi: 10.1016/j.jbc.2024.107557 (PMC11359735; doi:10.1016/j.jbc.2024.107557)
Supplement: Supporting information [file mmc1.pdf]

Table S1: Mining of transcriptome and proteome databases (https://plasmodb.org) for presence of GPI anchor biosynthesis machinery and GPI transamidase.

Gene Name	Oocyst Spz Transcriptome	Salivary Gland Spz Transcriptome	Salivary Gland Spz Proteome	Blood Stage Transcriptome	Blood Stage Proteome
**PF3D7_1032400 PIGA** phosphatidylinositol N-acetyl glucosaminyltransferase subunit A	Yes	Yes	No	Yes	No
**PF3D7_1141400** phosphatidylinositol N-acetyl glucosaminyltransferase subunit H	Yes	No	No	Yes	Yes
**PF3D7_0618900** phosphatidylinositol N-acetyl glucosaminyltransferase subunit GPI1	Yes	Yes	No	Yes	Yes
**PF3D7_0911000** phosphatidylinositol N-acetyl glucosaminyltransferase subunit C	Yes	Yes	No	Yes	No
**PF3D7_0935300** phosphatidylinositol N-acetyl glucosaminyltransferase subunit P	Yes	Yes	No	Yes	No
**PF3D7_0624700** N-acetylglucosaminyl-phosphatidylinositol de-N-acetylase	Yes	Yes	No	Yes	Yes
**PF3D7_0615300** GPI-anchored wall transfer protein 1, (inositol acyltransferase)	Yes	Yes	No	Yes	No
**PF3D7_1210900** GPI mannosyltransferase 1	Yes	Yes	No	Yes	No
**PF3D7_1247300** GPI mannosyltransferase 2	Yes	Yes	No	Yes	No
**PF3D7_1341600** GPI mannosyltransferase 3	Yes	Yes	No	Yes	No
**PF3D7_1214100** GPI ethanolamine phosphate transferase 3	Yes	Yes	Yes	Yes	Yes
**PF3D7_1122100** GPI transamidase component GPI16	Yes	No	Yes	Yes	Yes
**PF3D7_1330700** GPI transamidase subunit PIG-U	Yes	Yes	No	Yes	Yes
